# Diminished rostral anterior cingulate cortex activation during trauma-unrelated emotional interference in PTSD

**DOI:** 10.1186/2045-5380-3-10

**Published:** 2013-05-14

**Authors:** Reid Offringa, Kathryn Handwerger Brohawn, Lindsay K Staples, Stacey J Dubois, Katherine C Hughes, Danielle L Pfaff, Michael B VanElzakker, F Caroline Davis, Lisa M Shin

**Affiliations:** 1 Department of Psychology, Tufts University, Medford, MA, USA; 2 Department of Psychiatry, Massachusetts General Hospital, Charlestown, MA, USA

**Keywords:** fMRI, Stroop, Posttraumatic stress disorder, Anterior cingulate, Interference, Trauma

## Abstract

**Background:**

Previous research suggests that individuals with posttraumatic stress disorder (PTSD) preferentially attend to trauma-related emotional stimuli and have difficulty completing unrelated concurrent tasks. Compared to trauma-exposed control groups, individuals with PTSD also exhibit lower rostral anterior cingulate cortex (rACC) activation during tasks involving interference from trauma-related stimuli. However, it is not clear whether relatively diminished rACC activation in PTSD also occurs during interference tasks involving trauma-*unrelated* emotional stimuli. The present study employed functional magnetic resonance imaging (fMRI) and an interference task that involves emotional facial expressions and elicits rACC activation in healthy participants.

**Findings:**

While performing a trauma-unrelated emotional interference task, participants with PTSD (n=17) showed less rACC activation than trauma-exposed non-PTSD (TENP; n=18) participants. In the PTSD group, rACC activation was negatively correlated with the severity of re-experiencing symptoms. The two groups did not significantly differ on behavioral measures (i.e., response times and error rates).

**Conclusions:**

These findings suggest that relatively diminished rACC activation in PTSD can be observed in interference tasks involving trauma-unrelated emotional stimuli, indicating a more general functional brain abnormality in this disorder. Future neuroimaging studies need not employ trauma-related stimuli in order to detect rACC abnormalities in PTSD.

## Background

Posttraumatic stress disorder (PTSD) is a debilitating psychiatric disorder that occurs in 9-20% of individuals following a traumatic event [1]. Those with PTSD have difficulty suppressing trauma-related thoughts and suffer from persistent re-experiencing symptoms [2,3]. Such symptoms have been studied in the laboratory with interference tasks in which participants are asked to ignore trauma-related information (e.g., words) while performing an unrelated cognitive task (such as naming the color or number of the words). Indeed, individuals with PTSD have difficulty performing such tasks and show impaired behavioral performance (e.g. [3-5]) and relatively diminished activation in the rostral anterior cingulate cortex (rACC) [6,7]. The rACC is a brain structure that is thought to resolve emotional conflict [8] and/or “decrease the ‘weighting’ of affective information in the service of optimizing cognitive performance” [9]. Relatively diminished rACC function in PTSD may reflect a failure to (1) appropriately “weight” distracting emotional information and (2) inhibit the amygdala (e.g. [10]). In fact, activation of the rACC has been found to negatively correlate with PTSD symptom severity (e.g. [11,12]).

Previous neuroimaging studies that found reduced rACC activation in PTSD during interference tasks have used trauma-related words as stimuli [6,7]. If the same rACC abnormality could be demonstrated using trauma-*unrelated* emotional stimuli, it would suggest the presence of a more global emotional and attentional dysfunction in PTSD, as opposed to a deficit specific to trauma-related stimuli. Two recent studies examined rACC activation in participants with PTSD during trauma-unrelated emotional interference [13,14] but yielded conflicting results.

In the current experiment, we used functional magnetic resonance imaging (fMRI) to study rACC activation in individuals with PTSD and trauma-exposed non-PTSD (TENP) participants during the performance of an interference task using trauma-unrelated emotional stimuli (i.e., faces and words) [8,15]. We hypothesized that the PTSD group would exhibit diminished rACC activation relative to the TENP group when comparing high-interference to low-interference conditions. Given previous findings (e.g., [11-13]), we also hypothesized that, for the PTSD group only, rACC activation would inversely correlate with PTSD symptom severity.

## Methods

We recruited 42 right-handed participants who had been exposed to criterion A traumatic events (e.g., assault, motor vehicle accidents, abuse, and witnessing serious injury/death). Of these, 21 had current PTSD and 21 never developed PTSD. Within the PTSD group, two participants were removed due to excessive movement during the scan, one participant stopped the scan before the task began, and one participant failed to respond to an adequate number (75%) of trials. Within the TENP group, two participants were excluded due to a button box malfunction, and one participant was excluded due to excessive errors (greater than 25%). A total of 17 PTSD (14 female) and 18 TENP (13 female) participants were included in the final analyses.

PTSD diagnoses were determined using the Clinician Administered PTSD Scale (CAPS) [16]. All other diagnoses were made using the Structured Clinical Interview for the DSM-IV Axis I Disorders (SCID) [2]. Participants were free of the following: contraindications to MRI (e.g., metallic implants), complicating major medical conditions such as neurological disorders, pregnancy, current use of psychotropic medications, and a history of drug/alcohol abuse in the last six months. None of the TENP participants met criteria for any psychiatric disorders. Some participants with PTSD were also diagnosed with current major depressive disorder (MDD; n=5), specific phobia (n=1), and panic disorder (n=3). Psychometric and clinical data are presented in Table 1. All participants provided written informed consent. This study was in compliance with the Helsinki Declaration and approved by the institutional review board at Partners Healthcare System, Boston, Massachusetts.

**Table 1:** Demographic and psychometric data

**Variable**	**PTSD Mean (SD)**	**TENP Mean (SD)**	**Significance**
Age	29.88 (7.8)	27.06 (6.0)	*p*=0.235
Education	14.94 (2.4)	16.11 (2.1)	*p*=0.129
BMI	25.44 (4.6)	23.43 (3.4)	*p*=0.150
Current CAPS*	60.31 (15.1)	1.89 (3.1)	*p*<0.001
BDI*	13.12 (10.6)	1.27 (1.4)	*p*<0.001
BAI*	15.00 (9.5)	2.11 (3.6)	*p*<0.001

An asterisk (*) denotes a significant difference between groups. *BMI*, Body mass index; *CAPS*, Clinician administered PTSD scale; *BDI*, Beck depression inventory [17]; *BAI*, Beck anxiety inventory [18]; *SD*, Standard deviation.

### Stimuli and procedures

**Figure 1:**
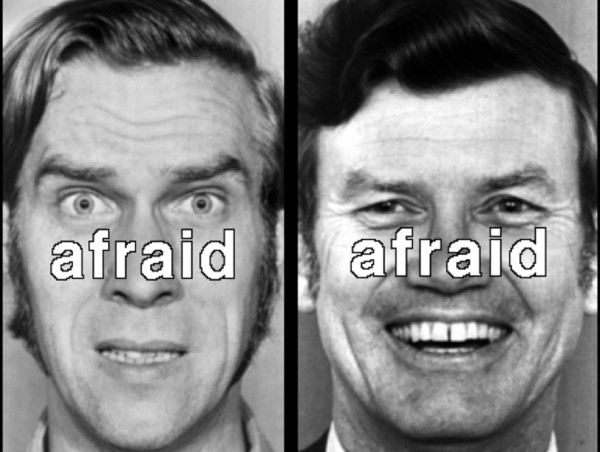
**The face on the left is an example of a Congruent trial, in which the word matched the facial expression.** The face on the right is an example of an Incongruent trial, in which the word did not match the facial expression.

Stimuli consisted of black and white photographs of 10 faces (5 male), each displaying one happy and one fearful expression [19], with either the word “happy” or “afraid” superimposed on each face (Figure 1). In the Congruent condition (low-interference), the superimposed word matched the facial expression. In the Incongruent condition (high-interference), the superimposed word did not match the facial expression. In the Baseline condition, a string of Xs was superimposed on the facial expression. Presentation timing was jittered using Optseq [20]. Faces were presented for 1300 milliseconds (ms), with a 700 ms inter-stimulus interval, in a pseudorandom order such that the same identity was never presented in succession. Fearful and happy faces were presented an equal number of times for a total of 180 stimuli per run. Interleaved within the facial stimuli were 28 white fixation crosses (null trials), which were presented for either 1300 ms or 3300 ms.

Participants completed four runs (6 minutes and 40 seconds each), but fMRI (and behavioral) analyses included only the first two runs to avoid decrements in anterior cingulate activation that are known to occur with extended task performance [21]. Face stimuli were displayed using MacStim Carbon 3.2.1 and projected via a Sharp Notevision6 (XG-NV6XU) LCD projector (Osaka, Japan). Participants used a button box to indicate whether faces were happy or afraid. Button assignments for “happy” and “afraid” were counterbalanced across subjects.

#### Image acquisition

Participants were scanned using a Symphony/Sonata 1.5T whole body high-speed imaging device, equipped for echo planar imaging (Siemens Medical Systems, Iselin, NJ) with a 3-axis gradient head coil. First, we collected an automated scout image and shimmed [22]. Next, we collected two high-resolution three-dimensional magnetization prepared rapid acquisition gradient echo (MPRAGE) sequences (TR/TE/Flip angle=2730 ms/3.39 ms/7°) with 1.33 mm slice thickness. We acquired fMRI blood-oxygen-level dependent (BOLD) signal images [23] using gradient echo T2*-weighted sequences (TR/TE/Flip angle=2000 ms/40 ms/90°) in 22 coronal slices, (thickness=7 mm, 1 mm gap), with interleaved excitation order and foot-to-head phase encoding. To allow longitudinal magnetization to reach equilibrium, four images were acquired and discarded before the start of each functional scan.

### Data analysis

#### Behavioral analyses

Response times were averaged across correct trials within each condition. Separate 2 (Group: PTSD, TENP) × 2 (Condition: Incongruent, Congruent) analyses of variance (ANOVA) were used to analyze response time and error rate data. With regard to error rates, we examined both errors of commission (incorrect response) and errors of omission (failure to respond) in separate ANOVAs.

#### FMRI analyses

We performed all fMRI statistical analyses using SPM 2.0 software (Wellcome Department of Cognitive Neurology, London, UK). Functional images were slice-time and motion corrected (realigned to the first volume in the time series), co-registered to structural images, spatially normalized into a standard stereotactic space (Montreal Neurological Institute [MNI] template), and smoothed with a Gaussian filter set at 7mm full width at half maximum.

Voxelwise Incongruent vs. Congruent (IvC) contrast images were created for each participant. These contrast images were then submitted to a second-level random effects model to assess differences between groups (PTSD vs. TENP). Trials involving either errors of commission or omission were excluded from all contrasts.

Statistical parametric maps were inspected for activations in rACC and dorsal anterior cingulate cortex (dACC) that exceeded a significance threshold of *p<*0.001 (one-tailed), uncorrected. We included the dACC as a secondary region of interest because previous studies have shown relatively greater dACC activation in PTSD versus non-PTSD groups (e.g., [6,14,24,25]). Consistent with previous research, the rACC was defined as the region superior/anterior to the corpus callosum and inferior to the cingulate sulcus, with a y coordinate greater than +30 [26]. The dACC was defined as the region superior to the corpus callosum and inferior to the cingulate sulcus, with a y coordinate between 0 and +30 [25-27]. Activations observed outside of these predefined regions of interest (ROIs) were subjected to the more conservative threshold of *p*<0.00001, uncorrected. Data from clusters exceeding these thresholds were extracted using MarsBaR (MRC Cognition and Brain Sciences Unit, Cambridge, United Kingdom).

#### Voxelwise whole brain correlations

To assess the relationship between current CAPS scores and IvC signal change in the PTSD group, we conducted the following voxelwise whole brain correlational analyses. First, we computed the IvC contrast within each participant in the PTSD group and correlated those contrast images with total CAPS scores. Because IvC activation could be related differently to different types of PTSD symptoms, we also ran correlations with CAPS-B (re-experiencing), CAPS-C (avoidance/numbing) and CAPS-D (hyper-arousal) subscale scores. We then inspected our regions of interest for significant clusters using the same p-value thresholds as described above.

## Findings

**Figure 2:**
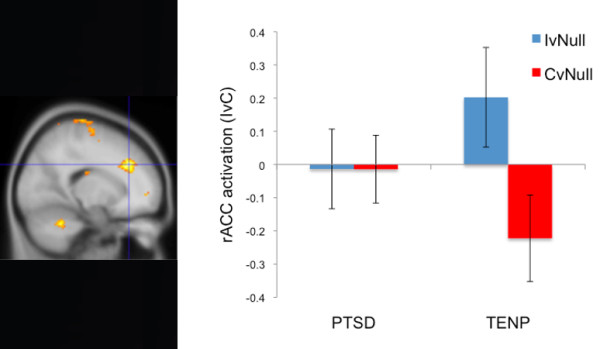
**The image on the left shows greater rACC (18, 32, 32) activation in the Incongruent versus Congruent (IvC) contrast in the trauma-exposed non-PTSD (TENP) group compared to the PTSD group.** The bar graph on the right breaks down this activation. IvNull shows fMRI signal in the Incongruent condition relative to the Null (focus cross) baseline. CvNull shows fMRI signal in the Congruent condition relative to the Null (focus cross) baseline. Error bars reflect standard error of the mean.

Significant main effects of Condition confirmed that response times and error rates were greater in the Incongruent vs. Congruent condition (Table 2). However, groups did not significantly differ on response time or error rate measures (*p*s > 0.32). Only the TENP group exhibited significant rACC activation in the IvC contrast (Table 3). When directly compared to the PTSD group, the TENP group exhibited significantly greater rACC activation (MNI x,y,z coordinates = 18, 32, 32) (Table 4). We extracted data from all significant voxels greater than z=3.09 (*p*<0.001) in this region for each condition compared to the null trials (i.e., fixation crosses). From these extractions, the TENP group exhibited activation during the Incongruent trials and deactivation during the Congruent trials. Comparatively, the participants of the PTSD group did not show such modulation of activation in the rACC (Figure 2). We also correlated the extracted data from the rACC (18, 32, 32) with symptom severity scores. Within the PTSD group, rACC activation was significantly negatively correlated with CAPS-B subscale scores (r [15]= −0.55, *p*=0.03) and had a trend-level negative correlation with total CAPS (r [15]= −0.44, *p*=0.08), but was not significantly correlated with other CAPS subscale scores, BAI scores, or BDI scores (all *p*s > 0.13). Within the TENP group, rACC activation did not significantly correlate with total CAPS scores, CAPS subscale scores, BAI scores, or BDI scores (all *p*s > 0.31).

**Table 2:** Response time (RT) and error rate (ER) results

	**PTSD (n=17)**				**TENP (n=18)**				**Mixed-Model ANOVA**^**a**^					
	**Congruent**		**Incongruent**		**Congruent**		**Incongruent**		**Group**		**Condition**		**Group x Condition**	
	**M**	**SD**	**M**	**SD**	**M**	**SD**	**M**	**SD**	**F**	***p***	**F**	***p***	**F**	***p***
RT (ms)	762	76	805	86	804	106	847	111	1.69	.20	102.7	<.001	.016	.89
ER (%)	.76	.74	2.87	2.36	.91	1.12	2.26	2.66	.201	.65	21.20	<.001	1.01	.32

Note that ^a^df = 1, 33 for all effects. *ms*, Milliseconds. *RT*, Response time; *ER*, Percent error.

**Table 3:** Incongruent vs. Congruent (IvC) contrast within PTSD and TENP groups

** Activation in PTSD group**			** Activation in TENP group**		
***Region***	***MNI (x,y,z)***	***z Score***	***Region***	***MNI (x,y,z)***	***z Score***
rACC	6, 32, 36	2.97 (ns)	rACC	16, 36, 34	4.48
dACC	12, 4, 42	3.40		−4, 34, 42	3.27
				16, 46, 2	3.23
				−10, 42, 32	3.10
			dACC	−12, 4, 44	3.96
				−14, 20, 32	3.33

There were no suprathreshold regions of activation for the inverse (CvI) contrast.

**Table 4:** Between-group comparison of the IvC contrast

**Activation greater in PTSD group**			**Activation greater in TENP group**		
***Region***	***MNI (x,y,z)***	***z Score***	***Region***	***MNI (x,y,z)***	***z Score***
-	-	-	rACC	18, 32, 32	3.57

**Figure 3:**
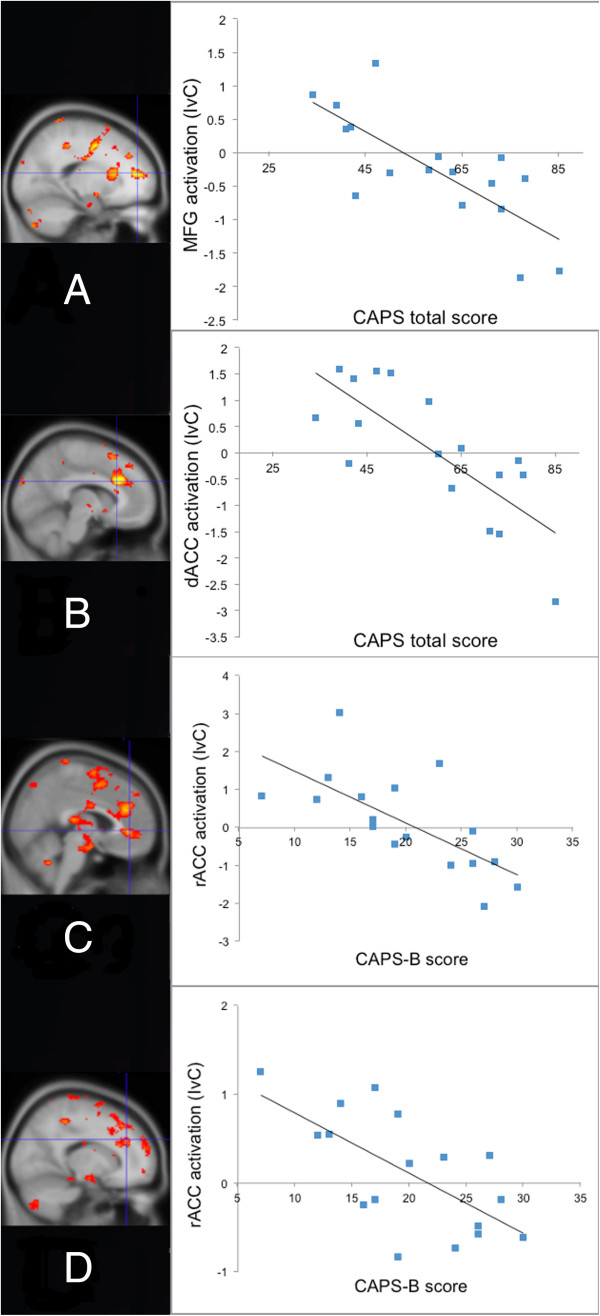
**In the PTSD group for the IvC contrast, total CAPS symptom severity scores negatively correlated with activation in the (A) medial frontal gyrus (MFG) (−18, 48, 14) and (B) dorsal anterior cingulate cortex (dACC) (−8, 24, 28).** In addition, CAPS-B scores (re-experiencing symptom severity) negatively correlated with rACC activation at (**C**) 4,40,0 and (**D**) 16,36,30.

Voxelwise correlation analyses revealed no significant negative correlation between total CAPS score and rACC activation (IvC) in the PTSD group; however, we did find a significant negative correlation between total CAPS scores and activation of medial frontal gyrus (MFG), just adjacent to the rACC (MNI x,y,z coordinates = −18, 48, 14; z=3.66; Figure 3A), and activation in a more dorsal region of the anterior cingulate cortex (dACC) (MNI x,y,z coordinates = −8, 24, 28; z=3.74; Figure 3B). We also ran voxelwise correlations between IvC and CAPS-B, C and D subscale scores in the PTSD group. The results were similar to those of the correlations with total CAPS scores with one important exception: only CAPS-B (re-experiencing) subscale scores were significantly negatively correlated with activation in the rACC proper (MNI x,y,z coordinates = 16, 36, 30; z=3.28 and 4, 40, 0; z=3.28; Figure 3C and D).

## Discussion and conclusion

In support of our primary hypothesis, the PTSD group exhibited lower rACC activation to trauma-*unrelated* interference, as compared to the TENP group. These results are consistent with those of Kim and colleagues [13] and suggest that diminished rACC function may reflect a more general abnormality in processing emotional material in PTSD.

We did not find any significant between-group differences on response times or error rates. It is possible that rACC dysfunction in PTSD is not behaviorally apparent until stimuli are more salient (trauma-related). Indeed, a recent meta-analysis has suggested that trauma-unrelated emotional word stimuli do not elicit greater behavioral interference in PTSD participants, as compared to controls [28]. However, previous studies that used trauma-related stimuli also failed to find significant behavioral impairment despite showing diminished rACC activation in PTSD [6,7]. It should be noted, however, that the latter study measured only accuracy (not response times) and the former study did report greater group differences in behavioral interference than the current study. Thus, it remains possible that behavioral evidence of rACC dysfunction is stronger when the stimuli are more salient. Regardless, the lack of behavioral differences in the present study suggests that our neuroimaging results cannot be attributed to group differences in behavioral performance.

Consistent with previous research, we found a trend toward a negative correlation between total symptom severity and activation in the rACC proper [11-13]. We also found significant negative correlations between total symptom severity and activation of structures adjacent to the rACC: the MFG and the dACC. Previous studies have revealed negative correlations between symptom severity and MFG activation (e.g., [29,30]). The observed dACC cluster lies 6mm away from the rACC boundary as defined herein. Given the smoothing kernel (7 mm), this cluster could reflect function of the rACC. Importantly, we also found that only re-experiencing symptom severity (CAPS-B) was significantly negatively correlated with activation in rACC proper. That the degree of diminished rACC activation during emotional interference may reflect the severity of re-experiencing symptoms per se makes sense given that emotional interference tasks were originally intended to tap into the cognitive processes underlying re-experiencing symptoms of PTSD [3].

If the rACC deficit in PTSD is more general (i.e., not specific to trauma-related material), then rACC activation to emotional interference should correlate negatively with PTSD symptom severity regardless of whether the emotional stimuli are trauma-related. Consistent with this hypothesis, Kim et al. [13] found that rACC activation to trauma-unrelated emotional stimuli is negatively correlated with PTSD symptom severity. In addition, we found a similar negative correlation in the current study between rACC activation and CAPS-B re-experiencing symptoms. Unfortunately, correlations between brain activation and symptom severity were not reported in other previous neuroimaging studies of emotional interference in PTSD [6,7,14].

In summary, our findings suggest that relatively diminished rACC function in PTSD may reflect a more generalized abnormality that can be observed even when emotional stimuli are unrelated to trauma. This finding also has implications for the planning of future neuroimaging studies that examine rACC function in groups with diverse trauma histories. Individually tailoring stimuli to match each participant’s traumatic event may not be necessary as emotional interference tasks need not contain trauma-related stimuli in order to reveal rACC abnormalities in PTSD.

## Abbreviations

ANOVA: Analysis of variance; BOLD: Blood oxygenation level dependent; CAPS: Clinician administered PTSD scale; dACC: Dorsal anterior cingulate cortex; DSM-IV: Diagnostic and statistical manual of mental disorders, fourth edition; fMRI: Functional magnetic resonance imaging; IvC: Incongruent versus Congruent contrast; MDD: Major depressive disorder; MFG: Medial frontal gyrus; MRI: Magnetic resonance imaging; PTSD: Posttraumatic stress disorder; rACC: Rostral anterior cingulate cortex; ROIs: Regions of interest; SCID: Structured clinical interview for the DSM-IV; TENP: Trauma-exposed non-PTSD.

## Competing interests

The authors declare that they have no competing interests.

## Authors’ contributions

RO collected and analyzed the data and drafted the manuscript. KHB collected and analyzed the data and edited the manuscript. LKS, SJD, KCH, DLP, MBV, and FCD assisted with data analysis and edited the manuscript. LMS designed and piloted the task, collected and analyzed the data, and edited the manuscript. All authors read and approved the final manuscript.
